# Effects of PDE-3 inhibition in persistent post-traumatic headache: evidence of cAMP-dependent signaling

**DOI:** 10.1186/s10194-024-01762-x

**Published:** 2024-04-17

**Authors:** Haidar M. Al-Khazali, Rune H. Christensen, Basit Ali Chaudhry, Anna G. Melchior, Messoud Ashina, Rami Burstein, Håkan Ashina

**Affiliations:** 1 Harvard Medical School, Boston, MA USA; 2 Department of Neurology, Beth Israel Deaconess Medical Center, Boston, MA USA; 3 Department of Neurology, Danish Headache Center, Copenhagen University Hospital– Rigshospitalet, Copenhagen, Denmark; 4 Department of Clinical Medicine, Faculty of Health and Medical Sciences, University of Copenhagen, Copenhagen, Denmark; 5 Department of Anesthesia, Critical Care and Pain Medicine, Beth Israel Deaconess Medical Center, Boston, MA USA; 6 Valdemar Hansens, Vej 5, Entrance 1A, 2600 Glostrup, Denmark

**Keywords:** Headache, Pain, Nociception, cAMP-Dependent signaling

## Abstract

**Background:**

Phosphodiesterase 3 (PDE-3) inhibition have been implicated in the neurobiologic underpinnings of migraine. Considering the clinical similarities between migraine and persistent post-traumatic headache (PPTH), we aimed to ascertain whether PDE-3 inhibition can elicit migraine-like headache in persons with PPTH.

**Methods:**

We tested cilostazol, which inhibits PDE-3, in a randomized, double-blind, placebo-controlled, two-way crossover study involving persons with PPTH attributed to mild traumatic brain injury. The randomized participants were allocated to receive oral administration of either 200-mg cilostazol or placebo (calcium tablet) on two separate experiment days. The primary end point was the incidence of migraine-like headache during a 12-hour observation window post-ingestion. The secondary endpoint was the area under the curve (AUC) for reported headache intensity scores during the same observation window.

**Results:**

Twenty-one persons underwent randomization and completed both experiment days. The mean participants’ age was 41.4 years, and most (*n* = 17) were females. During the 12-hour observation window, 14 (67%) of 21 participants developed migraine-like headache post-cilostazol, in contrast to three (14%) participants after placebo (*P* =.003). The headache intensity scores were higher post-cilostazol than after placebo (*P* <.001).

**Conclusions:**

Our results provide novel evidence showing that PDE-3 inhibition can elicit migraine-like headache in persons with PPTH. Given that PDE-3 inhibition increases intracellular cAMP levels, our findings allude to the potential therapeutic value of targeting cAMP-dependent signaling pathways in the management of PPTH. Further investigations are imperative to substantiate these insights and delineate the importance of cAMP-dependent signaling pathways in the neurobiologic mechanisms underlying PPTH.

**ClinicalTrials.gov Identifier:**

NCT05595993.

**Supplementary Information:**

The online version contains supplementary material available at 10.1186/s10194-024-01762-x.

## Introduction

Persistent post-traumatic headache (PPTH) represents a profound health burden, with disabling consequences often extending for years following mild traumatic brain injury (mTBI) [1, 2]. The clinical features can mimic primary headache disorders, such as migraine and tension-type headache [3]. Its clinical and phenotypic similarities with migraine, in particular, are striking [3]. However, despite this overlap, the neurobiologic underpinnings of PPTH remain incompletely understood, and evidence-based treatments are entirely lacking [4, 5]. This void necessitates an exploration of novel molecular targets and signaling pathways that might inform the development of efficacious therapeutic interventions.

Among the potential targets, phosphodiesterase 3 (PDE-3) presents an intriguing avenue for exploration. PDE-3 is an enzyme that breaks down cyclic adenosine monophosphate (cAMP), a critical second messenger molecule that transmits signals inside cells [6, 7]. In the context of cephalic pain, cAMP-dependent signaling pathways have emerged as a pathogenic driver [8–11]. Experimental data have shown that administration of cilostazol (a PDE-3 inhibitor) upregulates intracellular levels of cAMP and induces migraine attacks in persons with migraine [9–11]. Thus, it raises the intriguing question whether PDE-3 inhibition influences the genesis of cephalic pain percepts in PPTH, given the unmet treatment needs for this disorder.

In this randomized, placebo-controlled study, we aimed to ascertain whether PDE-3 inhibition, via cilostazol administration, can induce migraine-like headache in persons with PPTH. The insights derived from this work is intended to improve our understanding PPTH pathogenesis and possibly propel the development of targeted and effective therapeutic interventions.

## Methods

This study was performed with approval from the relevant ethics committee (H*-*21,067,676) and in adherence with the Declaration of Helsinki. The study is registered with ClinicalTrials.gov (NCT05595993). We obtained written informed consent from all participants prior to commencing any study-related tasks or procedures. All participants were provided with reimbursement for their time and travel expenses related to the study participation. All authors contributed to drafting or revising the manuscript and provided final approval for this version to be published.

### Population

We included persons aged 18–65 years who were diagnosed with PPTH attributable to mTBI and reported a minimum of four monthly headache days in the 3-month period prior to enrollment. The diagnosis of PPTH had to fulfil the criteria laid out in the 3rd edition of the International Classification of Headache Disorders (ICHD-3) [12]. We excluded persons with any history of a headache disorder (apart from infrequent episodic tension-type headache) and multiple mTBIs. Also excluded were persons who had initiated, altered, or discontinued preventive headache medication for the 2-month period prior to enrollment. The eligibility of participants was reliant on self-reported data and corroborated using electronic medical records. The full list of inclusion and exclusion criteria is shown in Supplemental Tables 1–2.

### Design

This was a randomized, double-blind, placebo-controlled, two-way crossover study. The randomized participants were allocated to receive oral administration of either 200-mg cilostazol or placebo (calcium tablet) on two separate experiment days, with a minimum wash-out period of one week in between.

The dosage of cilostazol used was identical to the one applied in previous human experimental studies [8–11]. Of note, participants were rescheduled for another experiment day if they had taken acute headache medication within the 48-hour timeframe preceding the administration of cilostazol or placebo. Participants were also rescheduled if they reported migraine-like headache at the time of drug/placebo administration (i.e., baseline) or if they reported a baseline headache intensity of > 3 on an 11-point numeric rating scale. The randomization process, allocation concealment, and drug preparation were carried out by independent research staff.

### Procedures

On the first experiment day, upon arrival, trained personnel recorded demographic and clinical data using a semi-structured interview. This was followed by a physical and neurologic examination, and participants were informed about the potential for headache induction after cilostazol administration. No specific details were provided about its characteristics, timing, or duration to avoid expectancy effects.

The remaining procedures were identical on both experiment days. Participants were placed in a supine position, and administration of either cilostazol or placebo was done orally. To ensure detailed documentation of outcome data, adverse events, rescue medication, and hemodynamic measures (arterial blood pressure and heart rate), participants were provided with a 12-hour headache diary. The first diary entry was made at baseline (at the time of drug intake), and subsequent recordings were done at 10-minute intervals until 1-hour post-administration. After completion of the 1-hour in-hospital observation window, participants were discharged and instructed to continue completing the diary on an hourly basis until 12 h post-administration.

### Sample size calculations

The sample size was estimated based on comparing cilostazol and placebo with respect to the primary end point, the incidence of migraine-like headache during a 12-hour observation window post-ingestion. McNemar’s test was used, under the assumption of an incidence rate of 50% exclusively after cilostazol and 10% exclusively after placebo. Given these estimates, we calculated that 21 participants would provide us with 80% power at a one-sided significance level of 0.05.

### Statistical analysis

**Fig. 1:**
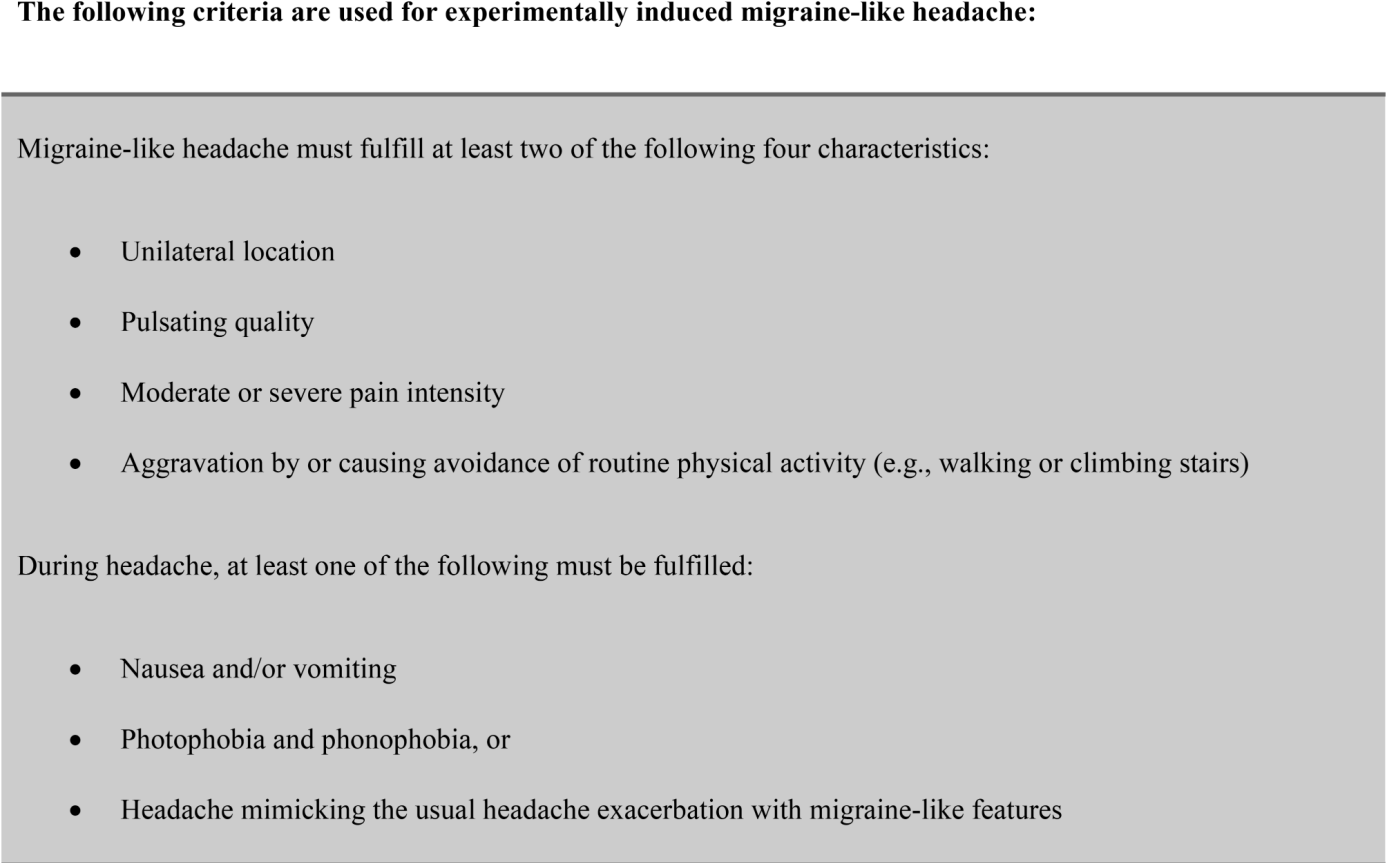
Criteria for Migraine-Like Headache

Descriptive statistics were applied to report on characteristics of the study population. We used means with standard deviations (SD) or medians with interquartile ranges (IQR) for continuous data, as appropriate. Categorical data was reported as absolute numbers accompanied by percentages. For the primary end point, we analyzed the outcome data using McNemar’s test. The criteria applied to define a migraine-like headache is shown in Fig. 1.

The secondary end point was the area under the curve (AUC) for headache intensity scores across the 12-hour observation window post-ingestion, comparing cilostazol and placebo. We applied the trapezium rule to calculate the AUC values and baseline-corrected the outcome data to minimize the influence of variations in headache intensities. This ensured a more accurate comparison of cilostazol and placebo with respect to the secondary end point, which was then analyzed using the Wilcoxon signed-rank test. Moreover, we calculated AUC values for the percentage change in mean arterial blood pressure and mean heart rate. Paired t-tests were then used to compare the hemodynamic responses between cilostazol and placebo. Lastly, we tested for carryover effects using a binomial regression model.

## Results

### Population

**Fig. 2:**
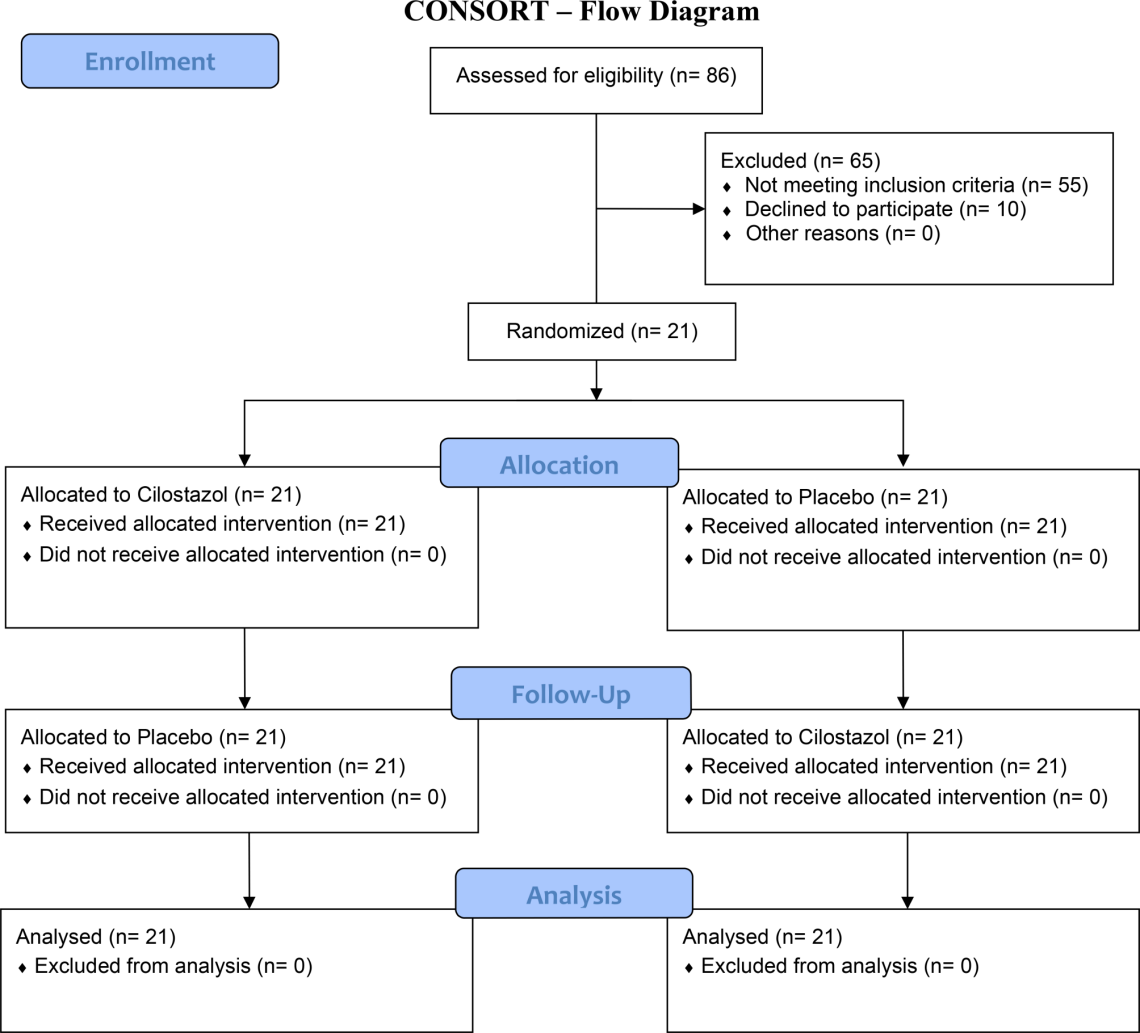
Consort Flow Diagram n, number

A total of 86 persons were screened, and 21 underwent randomization and completed the trial (Fig. 2 (Flow diagram)). The demographic and clinical characteristics of the participants are presented in Table 1. The participants were predominantly female (*n* = 17) and had a migraine-like phenotype (*n* = 19). The mean age was 41.4 (SD, 9.7) years, and the median duration since the sustained mild TBI was 10.1 (IQR, 6.6 to 10.6) years. Furthermore, the participants experienced a mean of 25.0 (SD, 8.0) monthly headache days, and nine reported ongoing use of preventive headache medications. A family history of migraine, with or without aura, was reported by 8 participants.

**Table 1:** Demographic and Clinical Characteristics of the Study Population

Characteristics	Persistent Post-Traumatic Headache, *n* = 21	
Age, mean ± SD, years	41.4 ± 9.7	
Body Mass Index, mean ± SD, kg/m^2^	26.3 ± 5.1	
Family History of Migraine without Aura, n (%)	6 (28.6)	
Family History of Migraine with Aura, n (%)	2 (9.5)	
Time since TBI, median (IQR), years	10.1 (6.6 to 10.6)	
Migraine-Like Phenotype, n (%)	19 (90.5)	
Tension-Type Headache-Like Phenotype, n (%)	2 (9.5)	
Monthly Headache Days, mean ± SD	25.0 (8.0)	
Current Use of Acute Headache Medication, n (%)	20 (95.2)	
Current Use of Preventive Headache Medication, n (%)	9 (42. 9)	

SD, standard deviation; IQR, interquartile range

### Headache response

**Fig. 3:**
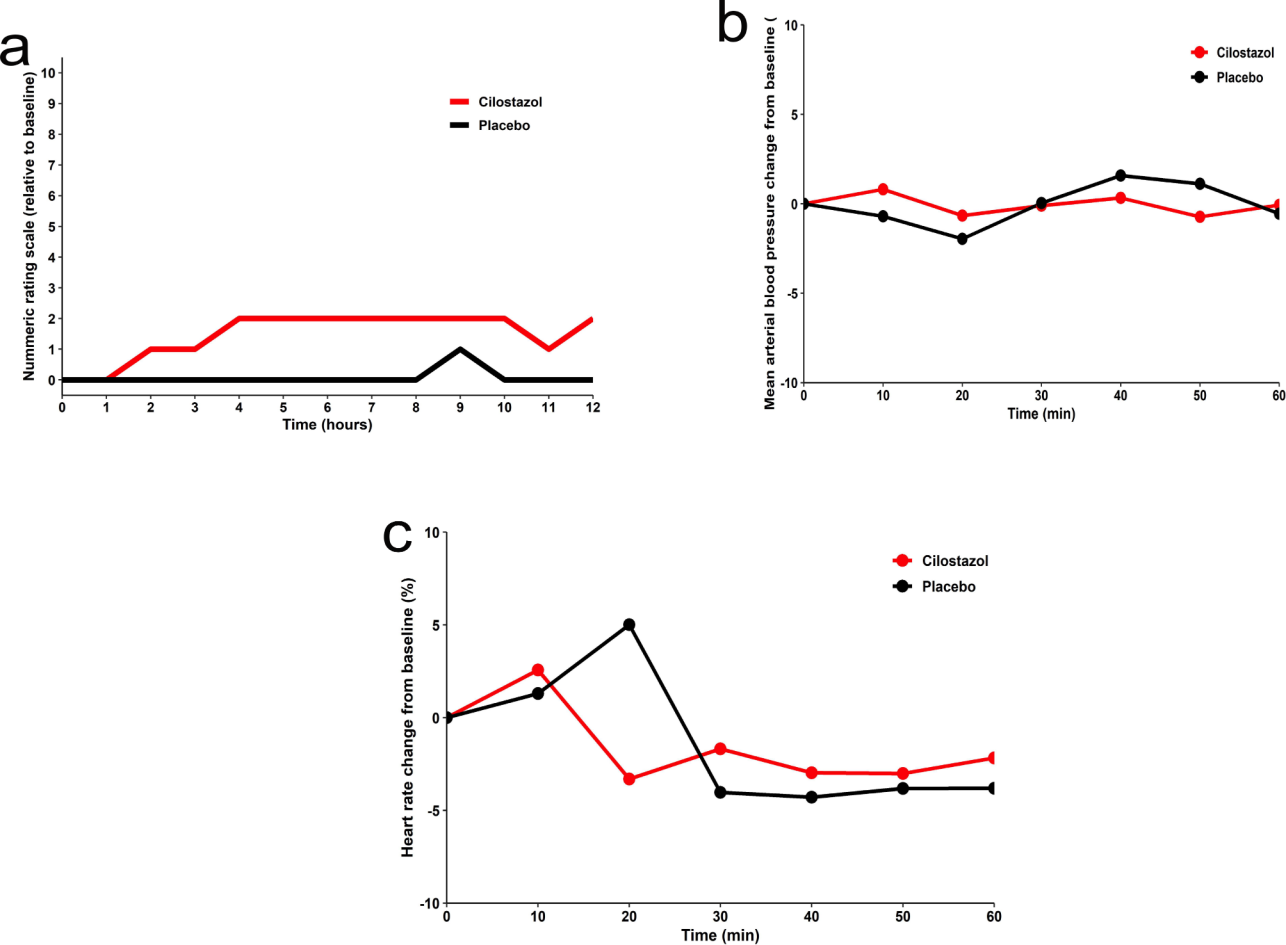
**a**. Baseline-Corrected Median Headache Intensity Scores after Cilostazol and Placebo. Median headache intensity scores, adjusted for baseline, following the administration of cilostazol and placebo during the 12-hour observational window. The baseline headache intensity scores were subtracted before calculating the area under the curve to minimize the impact of within-participant variations in headache at baseline between the two experimental sessions. The red line represents median headache intensity scores after cilostazol, while the black line signifies median headache intensity scores after placebo. **b**. Baseline-Corrected Mean Arterial Blood Pressures after Cilostazol and Placebo. Mean arterial blood pressure values, adjusted for baseline, following the administration of cilostazol and placebo within the 1-hour in-hospital observation window. The mean arterial blood pressure after cilostazol is represented by the red line, while the mean arterial blood pressure after placebo is depicted by the black line. **c**. Baseline-Corrected Mean Heart Rates after Cilostazol and Placebo. Mean heart rate values, adjusted for baseline, following the administration of cilostazol and placebo within the 1-hour in-hospital observation window. The mean heart rate after cilostazol is represented by the red line, while the mean heart rate after placebo is depicted by the black line.

During the 12-hour observation window, 14 (67%) of 21 participants experienced migraine-like headache after cilostazol, while three (14%) did so after placebo (*P* =.003, Supplemental Table 3). Eleven participants reported migraine-like headache exclusively after cilostazol, compared with none after placebo. No carryover effects were observed between cilostazol and placebo (*P* =.129). Furthermore, among participants who reported migraine-like headache, the median time to onset was 150 (IQR, 120 to 285) minutes after cilostazol and 420 (IQR, 300 to 540 min) minutes after placebo. The baseline-corrected headache intensity scores, expressed as AUC values, were higher following the administration of cilostazol, compared with placebo (*P <*.001; Fig. 3a). There were no carryover effects observed between cilostazol and placebo (*P* =.543). Furthermore, the median peak intensity reached a score of 6 (IQR, 5 to 7) after administration of cilostazol, compared with 4 (IQR, 2 to 5) post-placebo. The median change in headache intensity scores from baseline to the time of peak intensity was 4 (IQR, 3 to 6) after cilostazol and 2 (IQR, 0 to 2) after placebo.

Among the 21 participants, the phenotypic profile of the peak headache post-cilostazol was characterized primarily by a bilateral location (*n* = 18), a pressing quality (*n* = 12), and moderate-to-severe pain intensity (*n* = 19). Fourteen participants also experienced worsening of cephalic pain in relation to routine physical activity. Furthermore, accompanying symptoms included photophobia (*n* = 10), phonophobia (*n* = 11), and nausea or vomiting (*n* = 10).

### Adverse events, Rescue Medication, and hemodynamic measures

The most frequent adverse events were warm sensations (*n* = 7 after cilostazol, *n* = 4 after placebo), facial flushing (*n* = 3 after cilostazol, *n* = 4 placebo), and palpitations (*n* = 4 after cilostazol, *n* = 1 after placebo). Moreover, 11 of 21 participants took rescue headache medication after cilostazol administration, while three participants did so following placebo. In regard to hemodynamic measures, no differences were observed between cilostazol and placebo regarding mean arterial blood pressure (*P* =.91; Fig. 3b) and mean heart rate (*P* =.16; Fig. 3c) during the initial 1-hour in-hospital observation window.

## Discussion

In this randomized, placebo-controlled trial, we demonstrated that oral administration of cilostazol, a PDE-3 inhibitor, can elicit migraine-like headache in persons with PPTH, despite them having no pre-mTBI history of migraine. The spontaneous episodes of migraine-like headache experienced by the participants, closely resembled the characteristics of the migraine-like headache induced by cilostazol. Taken together, our results support the involvement of PDE-3 and cAMP-dependent signaling pathways in the pathogenesis of PPTH. Targeting these mechanisms might present novel avenues for drug development and fulfilling unmet treatment needs of persons with PPTH.

### PDE-3 inhibition, cAMP-Dependent signaling pathways, and Cephalic Pain

**Fig. 4:**
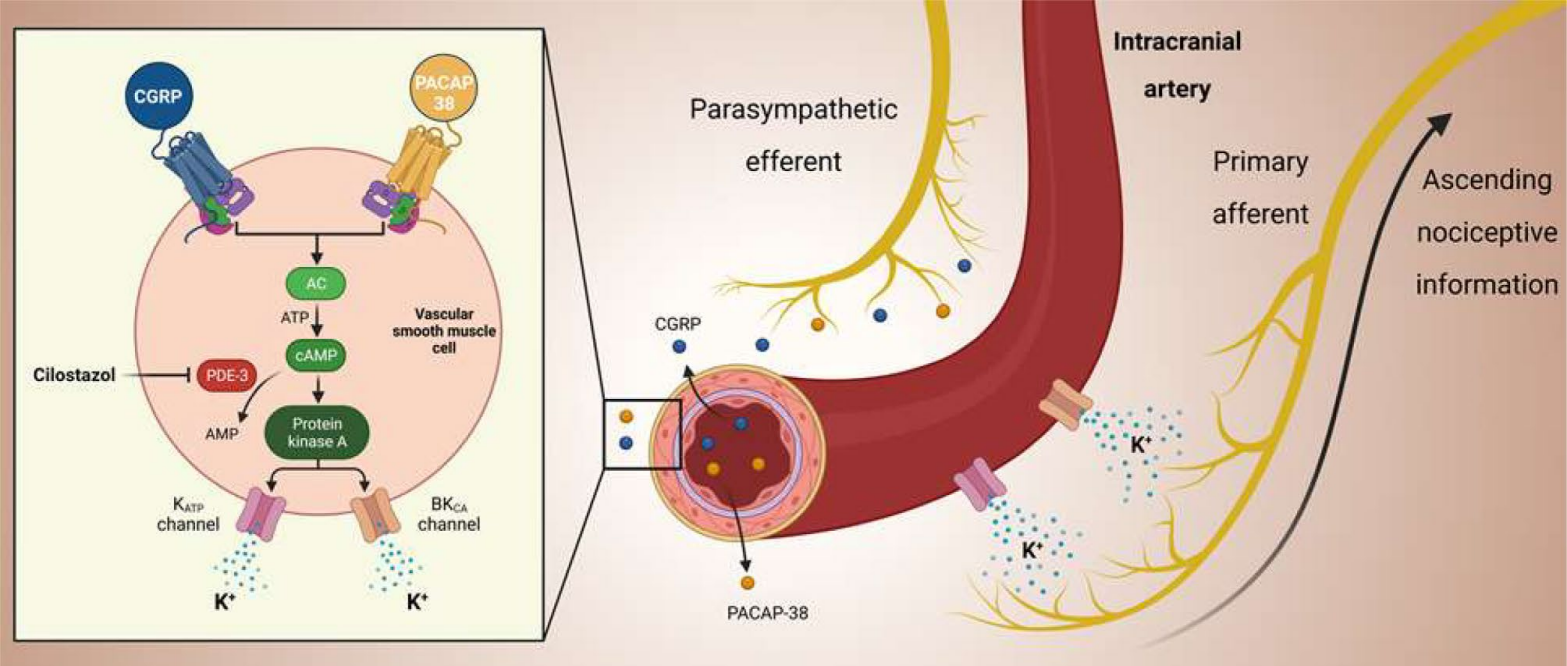
Proposed Mechanisms and Sites of Action of Cilostazol-Induced Migraine-Like Headache in Persons with PPTH. The illustration delineates a hypothesized mechanism and site of action whereby diverse pharmacological triggers contribute to the genesis of migraine-like headache in people with persistent post-traumatic headache (PPTH). In this proposed model, the neuropeptides calcitonin gene-related peptide (CGRP) and pituitary adenylate cyclase-activating polypeptide (PACAP-38) engage with their respective G protein-coupled receptors situated on vascular smooth muscle cells within the walls of meningeal arteries. Both peptides instigate adenylate cyclase (AC) activation via their transmembrane receptors, leading to an augmented intracellular cyclic adenosine monophosphate (cAMP) production. Cilostazol, a selective inhibitor of phosphodiesterase type 3 (PDE-3), impedes cAMP breakdown, resulting in its accumulation. The cAMP signaling pathway is postulated to activate and open ATP-sensitive potassium channels (KATP) and large conductance calcium-activated potassium channels (BKCa) channels. These subsequent events lead to potassium efflux, accompanied by the vasodilation of meningeal arteries. Modified from Al-Khazali et al., 2023.[19, 23]

The involvement of PDE-3 inhibition in cephalic pain has been well-documented in previous human experimental studies [8–11]. Oral administration of cilostazol has proven to elicit migraine attacks in persons with migraine [9–11], while causing mild headache in healthy volunteers [8]. It is also worth noting that cilostazol-induced migraine attacks are reproducible in persons with migraine, who were administered cilostazol on two separate experiment days [11]. Considering the evidence above and our own findings, it seems apparent that the pathogenesis of cephalic pain involves, at least in part, increased intracellular cAMP levels (Fig. 4).

To further substantiate the importance of cAMP-dependent signaling pathways, previous experimental studies have used calcitonin gene-related peptide (CGRP) and pituitary adenylate cyclase-activating polypeptide (PACAP) (Fig. 4) [13–16]. Both of these endogenous signaling molecules have been shown to induce migraine-like headache in persons with PPTH and migraine attacks in persons with migraine [15, 17–19]. Furthermore, the downstream effects of both CGRP and PACAP signaling converge on the upregulation of intracellular cAMP levels [13, 20]. This convergence provides yet another layer of evidence to support the assertion that targeting cAMP-dependent signaling pathways holds promise for developing mechanism-based drugs for PPTH.

### Possible sites and mechanisms of Action

The evidence discussed above has established that upregulating intracellular cAMP using PDE-3 inhibitors, such as cilostazol, elicits cephalic pain. However, important questions remain unanswered regarding the exact site(s) and mechanism(s) of action underlying this pro-nociceptive effect. One possible explanation involves a cascade of events, focusing on activation of perivascular meningeal nociceptors via mechanical and/or chemical stimulation [13, 20]. This hypothesis posits that the site of action for PDE-3 inhibitors is the vascular smooth muscle cells (VSMCs) within the walls of meningeal arteries [13, 20]. These arteries, located in the meninges that envelops the brain, regulate the cerebral blood flow. PDE-3 inhibitors exert their action by blocking the activity of PDE-3, an enzyme responsible for the breakdown of cAMP [6, 7]. This blockade leads to an accumulation of cAMP within the VSMCs, which facilitates the opening of two types of potassium channels– large conductance calcium-activated potassium (BK_Ca_) channels and ATP-sensitive potassium (K_ATP_) channels [21, 22]. Of note, recent experimental findings have demonstrated that pharmacologic opening of either potassium channel can elicit migraine-like headache in persons with PPTH [23]. Upon opening, both BK_Ca_ channels and K_ATP_ channels allow positively charged potassium ions to flow out of the VSMCs, causing them to hyperpolarize [13, 20]. This, in turn, leads to dilation of the meningeal arteries and possible mechanical stimuli, activating perivascular nociceptors [13, 20]. Furthermore, the increased levels of extracellular potassium might also contribute with chemical stimulation of the nociceptors. However, it merits emphasis that there is no firm evidence to confirm this sequence of events. It is, nonetheless, intriguing that all inducers of migraine-like headache in persons with PPTH are known to dilate meningeal arteries [18, 19, 23]. Findings from magnetic resonance angiography have further revealed that the middle meningeal artery is dilated exclusively on the pain-side at the onset time of cilostazol-induced migraine attacks [24]. This also aligns well with the reporting of cephalic pain after intraluminal balloon inflation of the middle cerebral artery [25]. Taken together, it seems timely for animal studies to ascertain whether dilation of meningeal arteries and potassium efflux from VSMCs can activate and sensitize meningeal nociceptors.

Another plausible site of action is the meningeal nociceptors themselves or their cell bodies located in the trigeminal ganglia and upper cervical dorsal root ganglia [26]. Preclinical studies have indicated that increased intracellular levels of cAMP can elicit activation of these ganglia neurons [27]. In support, application of prostaglandin E2 sensitizes cell culture with trigeminal ganglia neurons [28], as well as those with dorsal root ganglia neurons [29]. This finding is particularly relevant because prostaglandin E2 exerts its downstream effects through the upregulation of intracellular cAMP levels, and administration of prostaglandin E2 is known to trigger migraine attacks in persons with migraine [30]. This line of reasoning suggests that administration of cilostazol increases intracellular cAMP levels within the primary afferent nociceptive neuron. If true, the accompanying cilostazol-induced dilation of meningeal arteries would be unrelated to the pathogenesis of cephalic pain per se. The increased blood flow via vasodilation would then serve to meet the nociceptors’ increased metabolic demands.

In addition to potential peripheral sites of action, it is pertinent to consider the possibility that cilostazol might induce migraine-like headache a central site of action. There is indeed some evidence to support that cilostazol can impact the blood-brain barrier (BBB) [31]. Preclinical studies have shown that cilostazol can protect against BBB dysfunction in certain pathologic conditions, such as hemorrhagic stroke [31, 32]. This interaction with the BBB implies that cilostazol might exert a direct effect within the CNS. Nonetheless, it remains to be determined whether cilostazol’s pro-nociceptive effects are mediated at a central site of action, or if any central effects are consequential to its peripheral actions.

### Therapeutic implications and future directions

The therapeutic potential of PDE-3 activators for the treatment of PPTH is an emerging frontier in neuropharmacology. Our understanding of the role of PDE-3 and cAMP-dependent signaling pathways in the pathogenesis of headaches has evolved immensely in recent years. Experimental evidence, including the induction of cephalic pain in persons with PPTH after oral administration of cilostazol, underscores the pivotal role of PDE-3 and cAMP-dependent pathways in PPTH pathogenesis. Given this mechanistic insight, it is plausible to hypothesize that PDE-3 activation, leading to a decrease in intracellular cAMP levels, could potentially mitigate the symptoms of PPTH. This innovative approach could herald a novel therapeutic strategy for PPTH, a condition for which current treatment options are limited.

A key consideration in the development of PDE-3 activators is the specific targeting of VSMCs within the walls of meningeal arteries. This specificity is predicated on the understanding that the dilation of these arteries, a phenomenon associated with the activation of perivascular nociceptors and the onset of headaches, is modulated by the activity of PDE-3 in these cells [13, 20]. However, the path to developing PDE-3 activators as a viable treatment for PPTH is fraught with challenges [33]. Paramount among these is the need for selective targeting of PDE-3 in the affected tissues without disrupting cAMP signaling in other physiological systems [33]. This necessitates a granular understanding of the precise location and function of PDE-3 across different cell and tissue types [33]. Moreover, the development of PDE-3 activators must account for potential side effects, particularly in cells with high basal or stimulated cyclic nucleotide levels. It is also crucial to consider potential variations in the subcellular compartmentalization of PDE-3, which might differ across species, age, tissue types, and disease status.

Despite these challenges, the development of PDE-3 activators for the treatment of PPTH is an exciting and promising area of research. With continued studies to elucidate the precise role and regulation of PDE-3 in PPTH, coupled with advances in drug delivery systems, this approach could potentially revolutionize the therapeutic landscape for individuals suffering from this debilitating condition.

### Cilostazol-Induced Headache: a proof-of-Concept Model for Acute Headache medications

A recent innovative proof-of-concept model has involved using cilostazol-induced headache to test new drugs for treating acute headache. This concept rests on the assertion that if a drug can alleviate cilostazol-induced headache, it holds promise as an effective treatment for acute headache and merit further investigation in phase II/III trials. To validate this proof-of-concept model, two experimental studies used oral administration of sumatriptan, a well-documented acute headache medication, to treat cilostazol-induced headache [34, 35]. The first study used a double-blind, two-way crossover design, in which 30 healthy volunteers were randomly assigned to receive pre-treatment with sumatriptan or placebo on separate experiment days, followed by cilostazol [35]. The results showed no significant differences in headache intensity scores between sumatriptan and placebo groups at both 2- and 4-hours post-treatment. A similar experimental design was used in the second study, in which 30 adult participants with migraine were enrolled [34]. All participants received administration of oral cilostazol and were then randomly assigned to oral sumatriptan or placebo at 6 h post-cilostazol intake or upon the onset of moderate headache. The authors found no significant difference in headache intensity scores at 2 h post-treatment, but a significant difference was observed at 4 h, suggesting a delayed effect of sumatriptan. The collective findings from these studies suggest that cilostazol-induced headache is not a straightforward model for screening new acute headache medications. However, it is plausible that these studies were not adequately powered to detect small, yet clinically meaningful differences, between sumatriptan and placebo. In support, sumatriptan had a delayed effect at 4 h post-treatment in participants with migraine [34]. This highlights the need for further research with more robust experimental designs to ascertain whether cilostazol-induced headache is useful in a proof-of-concept model screening new acute headache medications.

### Limitations

Our study has some limitations that warrant some discussion. First, we limited the in-hospital observation window to 1 h due to logistic constrains. Environmental factors, such as dietary intake and stress, might therefore influence our findings. Second, we cannot exclude the impact of rescue medication use on the reported headache intensity scores. Third, we enrolled nine participants who reported using preventive headache medication. It is possible that this might influence the onset, duration, and characteristics of migraine-like headache induced by cilostazol. Fourth, our study population was predominantly female, and it cannot be excluded that the results might have been somewhat different in males. Lastly, epidemiologic data have raised intriguing questions about the relationship between migraine, head injuries, and PPTH. The available evidence suggests that head injuries are a risk factor for migraine chronification [36, 37], whilst pre-injury migraine is a risk factor for PPTH after mTBI [2, 38]. Thus, further research is needed to determine whether PPTH actually represents a migraine unmasked by trauma.

## Conclusions

Our discoveries delineate that PDE-3 inhibition elicits migraine-like headache in persons with PPTH. This accentuates the potential of targeting PDE-3 and cAMP-dependent signaling pathways as a novel therapeutic frontier for the effective management of PPTH. Thus, concerted research efforts are needed to unravel the role and involvement of PDE-3 and cAMP-dependent signaling pathways in the pathogenesis of PPTH.

### Electronic supplementary material

Below is the link to the electronic supplementary material.

Supplementary Material 1

Supplementary Material 2

## Data Availability

No datasets were generated or analysed during the current study.

## Abbreviations

PPTH: Persistent post-traumatic headache
mTBI: mild traumatic brain injury
PDE-3: phosphodiesterase 3
cAMP: cyclic adenosine monophosphate
ICHD-3: International Classification of Headache Disorders,3rd edition
SD: standard deviations
IQR: interquartile ranges
AUC: area under the curve
CGRP: calcitonin gene-related peptide
PACAP: pituitary adenylate cyclase-activating polypeptide
VSMCs: vascular smooth muscle cells
BK_Ca_: large conductance calcium-activated potassium channels
K_ATP_: ATP-sensitive potassium channels
