# Supplementary material for: Effects of PDE-3 inhibition in persistent post-traumatic headache: evidence of cAMP-dependent signaling
Source: J Headache Pain. 2024 Apr 17;25(1):56. doi: 10.1186/s10194-024-01762-x (PMC11022386; doi:10.1186/s10194-024-01762-x)
Supplement: Supplementary file 2 — Supplementary Material 2 [file 10194_2024_1762_MOESM2_ESM.docx]

**Supplemental Appendix.**

**Supplemental Table 1. Inclusion Criteria**

| Inclusion Criteria | Data Source |
| --- | --- |
| Age 18 to 65 years of age upon entry into screening | Legal identification document |
| History of persistent headache attributed to mild traumatic injury to the head for ≥ 12 months and in accordance with the International Classification of Headache Disorders, 3^rd^ Edition (ICHD-3) | Medical record and/or subject self-report as assessed by site investigator during the semi-structured interview |
| ≥ 4 monthly headache days on average across the 3 months prior to screening | Subject self-report as assessed by site investigator during the semi-structured interview |
| Provision of informed consent prior to initiation of any study-specific activities/procedures. | Informed consent form |

**Supplemental Table 2. Exclusion Criteria.**

| Exclusion Criteria | Data Source |
| --- | --- |
| > 1 mild traumatic injury to the head | Medical record and/or subject self-report as assessed by site investigator |
| History of any primary or secondary headache disorder prior to mild traumatic injury to the head (except for infrequent episodic tension-type headache) | Medical record and/or subject self-report as assessed by site investigator during the semi-structured interview |
| History of moderate or severe injury to the head | Medical record and/or subject self-report as assessed by site investigator during the semi-structured interview |
| History of whiplash injury | Medical record and/or subject self-report as assessed by site investigator during the semi-structured interview |
| History of craniotomy | Medical record and/or subject self-report as assessed by site investigator during the semi-structured interview |
| History or evidence of any other clinically significant disorder, condition or disease (except for those outlined above) than, in the opinion of the site investigator, would pose a risk to subject safety or interfere with study evaluation, procedures or completion | Medical record and/or subject self-report as assessed by site investigator during the semi-structured interview |
| The subject is at risk of self-harm or harm to others as evidenced by past suicidal behavior | Medical record and/or subject self-report as assessed by site investigator |
| Female subjects of childbearing potential with a positive pregnancy test during any study visit | Human chorionic gonadotropin (hCG) test (urine) |
| Cardiovascular disease of any kind, including cerebrovascular diseases | Medical record and/or subject self-report as assessed by site investigator during the semi-structured interview |
| Hypertension (systolic blood pressure of ≥150 mmHg and/or diastolic blood pressure of ≥100 mmHg) prior to the start of drug administration on the experimental day | Blood pressure measurement |
| Hypotension (systolic blood pressure of ≤90 mmHg and/or diastolic blood pressure of ≤50 mmHg) | Blood pressure measurement |
| Initiation, discontinuation, or change of dosing of prophylactic medications within 2 months prior to study inclusion | Medical record and/or subject self-report as assessed by site investigator during the semi-structured interview |
| Intake of acute medications (e.g. analgesics, triptans) within 48 hours of drug administration | Subject self-report as assessed by site investigator during the semi-structured interview |
| Baseline headache intensity of >3 on an 11-point numeric rating scale (0 being no headache, 10 being the worst imaginable headache) | Subject self-report as assessed by site investigator during the semi-structured interview |
| Baseline headache with migraine-like features or self-reported baseline headache that mimics the subjects’ usual headache with migraine-like features | Subject self-report as assessed by site investigator during the semi-structured interview |

**Supplemental Table 3. Clinical Characteristics of Study Participants after Cilostazol and Placebo.**

| Participant No./Sex | Headache Phenotype | Type of Intervention | Time to Peak Headache (min) | Peak Headache Characteristics^a^ | Mimics Usual Migraine-Like Headache^b^ | Migraine-Like Headache^c^ (Time to Onset) | Worsening of Associated Symptoms^d^ |
| --- | --- | --- | --- | --- | --- | --- | --- |
| **1/Male** | TTH-Like | Cilostazol  Placebo  Usual | 480 min  240 min | Bilat/6/Pres/+  Bilat/4/Pres/-  Bilat/5/Pres/- | No  No | Yes (120 min)  No | +/-/+  NA |
| **2/Female** | Migraine-Like | Cilostazol  Placebo  Usual | 300 min  40 min | Bilat/7/Throb/+  Bilat/4/Press/-  Bilat/8/Throb/+ | Yes  No | Yes (300 min)  No | +/+/+  NA |
| **3/Male** | Migraine-Like | Cilostazol  Placebo  Usual | 120 min  0 min | Bilat/10/Throb/+  Bilat/2/Throb/-  Unilat/8/ Throb/+ | Yes  No | Yes (120 min)  No | +/+/+  NA |
| **4/Female** | Migraine-Like | Cilostazol  Placebo  Usual | 240 min  0 min | Bilat/5/Pres/+  Bilat/2/Pres/-  Bilat/5/Throb/+ | No  Yes | No  No | NA  NA |
| **5/Female** | Migraine-Like | Cilostazol  Placebo  Usual | 120 min  180 min | Bilat/3/Pres/+  Bilat/2/Pres/-  Bilat/3/Throb/+ | No  No | No  No | NA  NA |
| **6/Female** | Migraine-Like | Cilostazol  Placebo  Usual | 240 min  240 min | Unilat/6/Throb/+  Unilat/8/Throb/+  Unilat/9/Throb/+ | Yes  Yes | Yes (180 min)  Yes (180 min) | +/+/+  +/+/+ |
| **7/Female** | Migraine-Like | Cilostazol  Placebo  Usual | 420 min  0 min | Bilat/5/Pres/+  Bilat/3/Pres/+  Unilat/6/Throb/+ | No  No | No  No | NA  NA |
| **8/Female** | Migraine-Like | Cilostazol  Placebo  Usual | 360 min  120 min | Unilat/7/Pres/+  Bilat/2/Pres/-  Unilat/7/Pres/+ | Yes  No | Yes (120 min)  No | -/+/+  NA |
| **9/Female** | Migraine-Like | Cilostazol  Placebo  Usual | 480 min  0 min | Unilat/3/Throb/+  Bilat/2/Pres/+  Bilat/8/Throb/+ | No  No | Yes (360 min)  No | +/+/+  NA |
| **10/Female** | Migraine-Like | Cilostazol  Placebo  Usual | 600 min  0 min | Bilat/9/Throb/+  Bilat/3/Pres/-  Unilat/5/Pres/ | Yes  No | Yes (30 min)  No | +/+/+  NA |
| **11/Female** | Migraine-Like | Cilostazol  Placebo  Usual | 360 min  50 min | Bilat/9/Throb/+  Bilat/5/Pres/-  Bilat/8/Throb/- | Yes  No | Yes (300 min)  No | +/-/-  NA |
| **12/Female** | Migraine-Like | Cilostazol  Placebo  Usual | 240 min  180 min | Bilat/5/Pres/+  Unilat/4/Throb/+  Unilat/4/Pres/ | No  No | Yes (240 min)  Yes (660 min) | -/+/+  -/+/+ |
| **13/Male** | Migraine-Like | Cilostazol  Placebo  Usual | 40 min  600 min | Bilat/7/Pres/+  Bilat/6/Pres/+  Bilat/8/Throb/+ | Yes  Yes | Yes (240 min)  Yes (420 min) | -/+/+  -/+/+ |
| **14/Female** | Migraine-Like | Cilostazol  Placebo  Usual | 30 min  240 min | Bilat/6/Throb/+  Bilat/5/Pres/-  Unilat/8/Throb/+ | Yes  No | Yes (30 min)  No | +/+/-  NA |
| **15/Female** | Migraine-Like | Cilostazol  Placebo  Usual | 600 min  480 min | Bilat/7/Pres/-  Unilat/4/Pres/-  Bilat/8/Pres/+ | Yes  No | No  No | NA  NA |
| **16/Female** | Migraine-Like | Cilostazol  Placebo  Usual | 540 min  300 min | Bilat/6/Pres/-  Bilat/4/Pres/-  Unilat/7/Pres/+ | No  No | No  No | NA  NA |
| **17/Male** | Migraine-Like | Cilostazol  Placebo  Usual | 300 min  300 min | Bilat/4/Pres/-  Unilat/6/Pres/-  Unilat/8/Throb/+ | No  No | No  No | NA  NA |
| **18/Female** | Migraine-Like | Cilostazol  Placebo  Usual | 120 min  360 min | Bilat/8/Throb/+  Unilat/4/Throb/+  Unilat/7/Throb/+ | Yes  No | Yes (120 min)  No | +/+/+  NA |
| **19/Male** | Migraine-Like | Cilostazol  Placebo  Usual | 300 min  600 min | Bilat/5/Throb/+  Bilat/3/Pres/-  Bilat/8/Comb/+ | No  No | Yes (300 min)  No | -/+/+  NA |
| **20/Female** | Migraine-Like | Cilostazol  Placebo  Usual | 360 min  0 min | Bilat/7/Pres/+  Unilat/1/Pres/-  Unilat/7/Pres/+ | No  No | Yes (240 min)  No | +/-/-  NA |
| **21/Female** | TTH-Like | Cilostazol  Placebo  Usual | 480 min  420 min | Bilat/5/Pres/+  Bilat/3/Pres/+  Bilat/4/Pres/- | No  No | No  No | NA  NA |

No, number; TTH, tension-type headache; Bilat, bilateral; Throb, throbbing; Unilat, unilateral; Pres, pressing: NA, not applicable; Comb, combined throbbing and pressing quality of headache.

a Localization (unilateral, bilateral) / pain intensity (11-point numeric rating scale, with 0 indicating no headache and 10 indicating the worst headache imaginable) / quality of headache (throbbing, pressing, combined throbbing and pressing) / aggravation of headache by routine physical activity (plus denotes presence, minus denotes absence).

b The participant is asked to determine whether the headache following Cilostazol or placebo intake resembles his/her, if applicable, usual migraine-like headache.

c Migraine-like headache is defined by the criteria outlined in Table 1.

Associated symptoms are nausea, photophobia, and phonophobia. Worsening is defined as an increase in the severity of these symptoms at the onset of migraine-like headache compared with baseline (i.e., time of drug intake start), as rated on a 4-point Likert scale (0 = none, 1 = mild, 2 = moderate, 3 = severe).
